# Emerging Retirement Crisis: When Social Security Is Not Enough

**DOI:** 10.1093/geroni/igaa057.2433

**Published:** 2020-12-16

**Authors:** David Miller

**Affiliations:** Case Western Reserve University, Cleveland, Ohio, United States

## Abstract

Evidence suggests a growing retirement crisis in the United States among older adults placing many of them at risk of falling into poverty. While Social Security provides income assistance to retirees, the average monthly benefit is $1,300. Among older adults nearing or in retirement, the use of public assistance programs is increasing. Using data collected by the Associated Press-NORC Center for Public Affairs Research we examine retirement preparedness, borrowing from retirement plans, and use of social welfare programs. Findings indicate increased borrowing from retirement plans due to debt, significant differences in racial and gender groups accessing and receiving services among those 75 and older. Increasing rates of unpreparedness for retirement exist among older Americans, particularly among adults of color. An increase in the use of safety net services among older adults is occurring concurrently with severe funding reductions in social welfare programming.

